# GC Method Validation for the Analysis of Menthol in Suppository Pharmaceutical Dosage Form

**DOI:** 10.1155/2017/1728414

**Published:** 2017-03-06

**Authors:** Murad N. Abualhasan, Abdel Naser Zaid, Nidal Jaradat, Ayman Mousa

**Affiliations:** ^1^Department of Pharmacy, Faculty of Medicine & Health Sciences, An Najah National University, Nablus, State of Palestine; ^2^R&D Department, Avalon Pharma, Riyadh, Saudi Arabia

## Abstract

Menthol is widely used as a fragrance and flavor in the food and cosmetic industries. It is also used in the medical and pharmaceutical fields for its various biological effects. Gas chromatography (GC) is considered to be a sensitive method for the analysis of menthol. GC chromatographic separation was developed using capillary column (VF-624) and a flame ionization detector (FID). The method was validated as per ICH guidelines for various parameters such as precision, linearity, accuracy, solution stability, robustness, limit of detection, and quantification. The tested validation parameters were found to be within acceptable limits. The method was successfully applied for the quantification of menthol in suppositories formulations. Quality control departments and official pharmacopeias can use our developed method in the analysis of menthol in pharmaceutical dosage formulation and raw material.

## 1. Introduction

**Figure 1:**
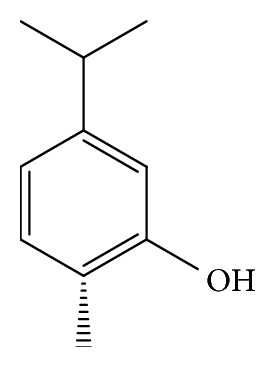
Menthol structure.

Menthol is a phytogenic essential oil that is considered as a monocyclic monoterpenoid alcoholic compound. The main form of menthol occurring in nature is (−)-menthol (Figure 1). It is isolated from various mint plants species such as* Mentha piperita*,* Mentha canadensis*,* Mentha arvensis,* and* Mentha spicata *[1]. Menthol can also be semisynthesized from other essential oils such as turpentine oil, eucalyptus oil, and citronella oil. Due to its peculiar and cooling properties, this natural compound has been used from centuries as a fragrance and flavor in the food and cosmetic industries [2, 3].

This natural compound is used widely in the medical and pharmaceutical fields for its biological effects such as analgesic, antifungal, antipruritic antibacterial, anticancer, and anti-inflammatory activities [4]. In addition, menthol is present in pharmaceutical preparations that are used as cooling agent in the counterirritant rubefacients, mouth and throat antiseptics, hemorrhoids, and many other pharmaceutical formulation [5–7]. Menthol is well known for its cooling sensation effect when it is chewed, inhaled, consumed, or applied to the skin due to its ability to chemically activate the cold sensations transient receptor potential cation channel [8].

Menthol is also present in many suppository dosage form and widely sold in the local and international markets. It is mainly indicated for the treatment of occasional minor irritation, pain, and cough associated with cold or inhaled irritants [9, 10].

Many methods have been established for the analysis of menthol, including HPLC methods with fluorescence-labeling reagents [11], refractive index [12], and polarized photometric detector [13]. Normal-phase HPLC with refractive index detector has also been employed in the analysis of menthol [14]. However, these methods have low sensitivity. Gas chromatography is considered to be a sensitive method for the analysis of menthol and it has been widely employed in the analysis of menthol in food and cosmetics [15]. To the best of our knowledge, there is not a specific analytical method that has applicability of analyzing menthol in actual pharmaceutical samples such as suppositories. Moreover, none of the most recognized pharmacopeias include the analysis of menthol in suppository dosage form.

The objective of our work was to establish a simple and rapid analysis of menthol in suppository pharmaceutical products by GC. The method was validated according the international guideline described in the ICH and international pharmacopeias [16]. The method was validated in terms of linearity, precision, accuracy, and ruggedness [17]. The method can be routinely used for the purpose of determination of menthol in formulated suppositories and in raw material.

## 2. Experimental

### 2.1. Chemicals

The analytical standard menthol (purity > 99.57%) was purchased from Frey & Lau GmbH, Immenhacken, Henstedt-Ulzburg, Germany; menthol reference standard was purchased from USP [Catalogue number 1381709]. Ethanol absolute was of GC grade from Thermo Fisher Scientific (Fair Lawn, New Jersey, USA).

Ultrapure water was obtained from Elga pure water system (Elga, model LA621, UK).

All other reagents were of pharmaceutical grade and used as received.

### 2.2. Chromatographic Conditions

Chromatographic separation was performed using capillary column, VF-624 ms (phase composition: 6% cyanopropylphenyl and 94% dimethylpolysiloxane), with film thickness of 1.8 *μ*m, and length of 60 m. The experiments were performed on Thermo GC Model (Trace Ultra Gas Chromatography, Thermo Fisher Scientific, USA) equipped with autosampler (Thermo Triplus) and a flame ionization detector (FID). The GC parameters are summarized in Table 1.

### 2.3. Preparation of Sample and Standard Solutions

The internal standard thymol (2% w/v) was prepared by dissolving in absolute ethanol. Standard solutions were prepared by weighing accurate weight of 30 mg of menthol working standards into 50 mL volumetric flask, adding 25 mL of ethanol, and sonicating for 5 minutes. 2.0 ml of the prepared Thymol Solution (Internal Standard) was added to it and the volume was completed to 25 ml with ethanol [18].

Sample preparation solutions were prepared by accurately weighing 3.0 g of smashed suppositories and were placed in 50 mL volumetric flask; then 5 mL of THF was added to it. The mixture was stirred vigorously for about 30 minutes on a shaker; then 2.0 mL of Thymol Solution (Internal Standard) was added to it and the volume was complete with diluents. The solution was filtered with cotton and with 0.22 *µ*m pore filter and then injected directly. The final solution of the sample was kept at room temperature to avoid precipitation.

### 2.4. Assay Calculation

The percentage assay of menthol was calculated using the following formula [19]:

(1) $$\begin{array}{c} Assay\;\;of\;\;methanol\;\;\%=\frac{Rsple*Wstd*P*100\%}{Rstd*Wsple*9.682}, \end{array}$$

where R*sple *** =** Ratio of the peak area of menthol divided by the peak area of the thymol in sample preparation. R*std* is the average ratio of the peak area of menthol divided by the peak area of the thymol in the standard preparations. W*std* is the weight of menthol working standard (mg). W*sple* is the weight taken in sample preparation (g). *P* is the purity of menthol working standard.

### 2.5. Method Validation

#### 2.5.1. System Suitability and Precision

The system suitability parameter for 10 replicate injections of Menthol and Thymol ratio was performed. The relative standard deviation (RSD) of ratio of the peak area of Menthol and Thymol of the replicate injections of standard solution should have an RSD not more than 2.0%.

#### 2.5.2. Linearity and Range

In order to evaluate the linearity of assay procedure, a series of standards at different concentrations of the target concentration for menthol was prepared in the range of 0.3–0.9 mg/mL which corresponds to 50%–150% relative to menthol measuring concentration in standard solution. After chromatographing each preparation in triplicate, a linear regression analysis was performed on the average peak ratio versus the concentrations of the levels studied.

The correlation coefficient was calculated by plotting component average peak ratio versus component concentrations. Linear regression was applied to the plots and the correlation coefficients for component data were calculated. In order for the test to pass the square of correlation coefficient should not be less than 0.998.

The limit of detection (LOD) and limit of quantification (LOQ) were calculated based on the standard deviation (s.d) of the data and the slop of the regression line. The LOD was calculated according to the following equation: 3.3 *∗* s.d /slope. The LOQ was calculated according to the following equation: 10 *∗* s.d/slope.

#### 2.5.3. Accuracy

The accuracy was performed based on three concentrations around the test concentration (80%, 100%, and 120%); three replicates of each concentration were injected. The percentage of recovery and percentage of RSD were calculated for each of the repeated samples.

#### 2.5.4. Method Precision

In order to evaluate the precision of assay method of menthol, six samples of suppositories were prepared and injected in replicate. The percentage of recovery and percentage of RSD were calculated for each of the repeated samples. The percentage of RSD must be less than 2.0 and all percentages of accuracy results must be within the specifications (within ±20.0% of the actual amount for menthol).

#### 2.5.5. Ruggedness of the Method

Ruggedness of the analytical method was performed by running samples in two days by different analysts, using different instruments. In order for the method to be rugged the percenage of RSD results between day 1 and day 2 must be less than or equal to 5 and all percentage of accuracy results must be within ±20.0% of the actual amount.

### 2.6. Stability of Analytical Solution

The stability of analytical solution was determined by analyzing menthol in both the standard solutions and sample solution over 24-hour period. The percentage of recovery of menthol was calculated versus the fresh injections for both standard solutions and sample solutions. In order to prove the stability of the solution the standard deviation of all the test solutions must be within ±2.0%.

## 3. Result and Discussion

### 3.1. System Suitability and Precision

It is generally desirable to ascertain the suitability and effectiveness of the operating system when employing chromatographic methods such as gas chromatography to ascertain the effectiveness of the final operating systems; it should be subjected to a suitability test prior to use. The essence of such test is the concept that the electronics, the equipment, the specimens, and the analytical operations constitute a single analytical system, which is amendable to an overall test of the system functions.

The result of system precision of 10 replicate injections shows that the RSD value was 1.071 (Table 2).

**Figure 2:**
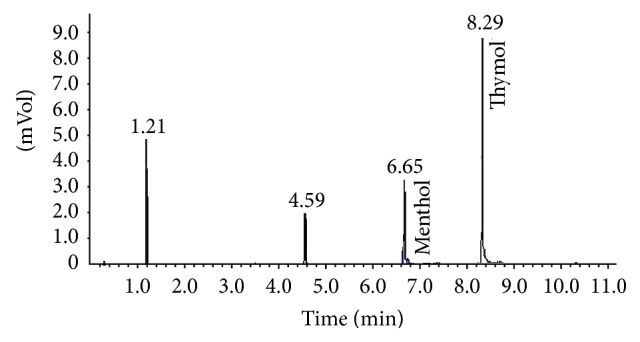
GC chromatogram of menthol and internal standard Thymol.

The GC chromatogram showed well separated peaks for menthol and the thymol internal standard (Figure 2).

### 3.2. Method Validation

#### 3.2.1. Linearity and Range

**Figure 3:**
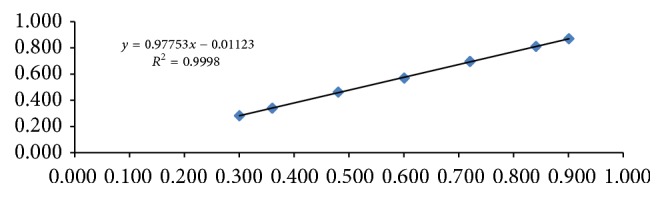
Linearity for menthol in suppository.

The correlation coefficient was calculated by plotting component average peak ratio versus component concentrations and was found to be 0.9998. Linear regression was applied to the plots and the slop was found to 0.9775 with an intercept of −0.0112 (Table 3 & Figure 3).

Limit of detection and limit of quantitation were calculated and found to be 0.01 mg/mL and 0.03 mg/mL, respectively.

#### 3.2.2. Accuracy

The purpose of this test was to prove accuracy of the method and show that none of the suppository excipients interfere with menthol active ingredients. The analytical methods were accurate, and the percentage of recovery for the all the tested samples was in the range of 98–102 and the RSD was less than 2. The test results are illustrated in Table 4.

#### 3.2.3. Method Precision

The method is precise and the percentage of recovery results for preparations is shown in Table 5 and the percentage of RSD of the tested samples was calculated and found to be 0.9.

#### 3.2.4. Ruggedness of the Method

The percentage of recovery results for twelve preparations was calculated and was found to be 105. The average percentage of RSD for day 1 and day 2 results was found to be 1.2. The method therefore is considered rugged.

### 3.3. Stability of Analytical Solutions

It is important to know if the solutions used in the analytical of both the standard solutions and the sample solutions are stable over time. This was determined by analyzing menthol over a period of 24 hours; the percentage of recovery was calculated versus the fresh injections for the standard solutions and sample solution. In order to prove the stability of the solution, the standard deviation for results should be within ±2.0%. Both the standard solution and the sample solution were stable for 24 hours and the calculated percentage of RSD for the test results was less than 0.5.

## 4. Conclusion

A new and suitable GC assay method utilizing FID detection has been developed for the analysis of menthol in pharmaceutical suppositories. This method is novel, economical, rapid, and specific for the assay of that active ingredient menthol. The developed method has been validated in accordance with both FDA and ICH guidelines and showed excellent linearity, accuracy, precision, and system suitability.

## Figures and Tables

**Table 1:** The GC parameters.

Carrier gas	Helium			
Column oven temperature (programmed)	Rate (°C/minute)	Temperature (°C)	Hold time (minute)	Total time (minute)
	Initial	90	1.0	1.0
	15	181	3.0	18.0
	Total Time			19.0
Detector temperature	180°C			
Injector temperature	280°C			
Flow rate	5.0 mL/ minute (constant)			
Split ratio	50			
Injection volume	1 *µ*L			
Gases flow rate	Makeup Gas			
	He → 30 mL/minutes			
	H2 → 30 mL/minutes			
	Air → 300 mL/minutes			

**Table 2:** System suitability and system precision result.

Injection number	Area of menthol (mVot)	Area of thymol (mVot)	Ratio
1	76756	124806	0.615
2	76491	124418	0.615
3	74717	122117	0.612
4	76281	125238	0.609
5	75446	123684	0.610
6	74402	121399	0.613
7	75591	124163	0.609
8	73692	121011	0.609
9	78280	125238	0.625
10	78409	125059	0.627

AVG	*76007*	*123713*	*0.614*

RSD%	*2.052*	*1.308*	*1.071*

**Table 3:** Linearity results for menthol.

Level #	Concentration (mg/mL)	Ratio
1	0.300	0.283
2	0.360	0.340
3	0.481	0.463
4	0.601	0.569
5	0.721	0.696
6	0.841	0.812
7	0.901	0.869

*R* ^2^	0.9998	

Slope	0.9775	

Intercept	−0.0112	

**Table 4:** Accuracy results for menthol.

^*∗*^Relative concentration%	Concentration (mg/mL)	Preparation #	Average % recovered
80%	0.48	1	99.5
		2	100.4
		3	101.3
		*Average*	*100.4*
		*% RSD*	*0.896*

100%	0.60	1	101.1
		2	98.4
		3	99.6
		*Average*	*99.7*
		*% RSD*	*1.370*

120%	0.72	*1*	101.3
		*2*	100.3
		*3*	100.1
		*Average*	*100.6*
		*% RSD*	*0.64*

^*∗*^Relative to menthol concentration in standard solution.

**Table 5:** Method precision results.

Preparation #	Rep #	Ratio	Assay
1	1	0.8102	104.9
	2	0.8113	105.0
2	1	0.8042	102.3
	2	0.8109	103.1
3	1	0.8150	104.0
	2	0.8129	103.7
4	1	0.9122	104.6
	2	0.9229	105.8
5	1	0.9018	104.1
	2	0.9083	104.8
6	1	0.8586	103.7
	2	0.8555	103.4
		*Average*	*104.1*
		*% RSD*	*0.9*
