# Corrigendum to “GC Method Validation for the Analysis of Menthol in Suppository Pharmaceutical Dosage Form”

**DOI:** 10.1155/2018/2561921

**Published:** 2018-08-13

**Authors:** Murad N. Abualhasan, Abdel Naser Zaid, Nidal Jaradat, Ayman Mousa

**Affiliations:** ^1^Department of Pharmacy, Faculty of Medicine & Health Sciences, An Najah National University, Nablus, State of Palestine; ^2^R&D Department, Avalon Pharma, Riyadh, Saudi Arabia

In the article titled “GC Method Validation for the Analysis of Menthol in Suppository Pharmaceutical Dosage Form” [1], the menthol structure presented in Figure 1 was incorrect and should be corrected as follows.

## Figures and Tables

**Figure 1:**
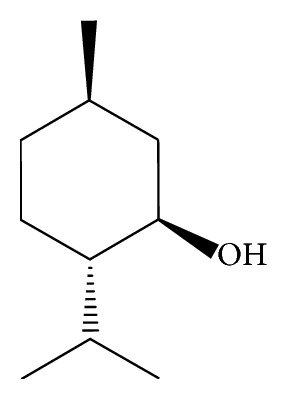
Structure of (–)-menthol.

## References

[1] Abualhasan M. N., Zaid A. N., Jaradat N., Mousa A. (2017). GC Method Validation for the Analysis of Menthol in Suppository Pharmaceutical Dosage Form. *International Journal of Analytical Chemistry*.

